# The Warthin-Like Variant of Papillary Thyroid Carcinoma: A Comparison with Classic Type in the Patients with Coexisting Hashimoto's Thyroiditis

**DOI:** 10.1155/2015/456027

**Published:** 2015-04-23

**Authors:** Min-kyung Yeo, Ja Seong Bae, Sohee Lee, Min-Hee Kim, Dong-Jun Lim, Youn Soo Lee, Chan Kwon Jung

**Affiliations:** ^1^Department of Hospital Pathology, College of Medicine, The Catholic University of Korea, Seoul 137-701, Republic of Korea; ^2^Department of Pathology, Chungnam National University School of Medicine, Daejeon 301-747, Republic of Korea; ^3^Department of Surgery, College of Medicine, The Catholic University of Korea, Seoul 137-701, Republic of Korea; ^4^Department of Internal Medicine, College of Medicine, The Catholic University of Korea, Seoul 137-701, Republic of Korea

## Abstract

*Background*. The Warthin-like variant of papillary thyroid (WLPTC) is a rare subtype of papillary thyroid carcinoma (PTC) resembling Warthin tumors of the salivary glands. Due to its rarity, the clinicopathologic and molecular features of WLPTC remain unclear. *Methods*. Of the 2,139 patients who underwent surgical treatment for PTC from 2012 to 2013, 40 patients with WLPTC were identified and compared to 200 consecutive patients with classic PTC. *BRAF* mutation was tested with pyrosequencing. *Results*. There were no significant differences in age, predilection for women, multifocality, extrathyroidal extension, or lymph node metastasis between WLPTC and classic PTC. However, WLPTCs were more commonly associated with Hashimoto's thyroiditis than classic PTCs (93% versus 36%, resp., *P* < 0.001) and showed significantly lower rate of *BRAF* mutation when compared to classic PTCs (65% versus 84%, resp., *P* = 0.007). In classic PTC, the frequency of *BRAF* mutations was negatively correlated with coexisting Hashimoto's thyroiditis. When we compared WLPTC and classic PTC in the patients with coexisting Hashimoto's thyroiditis, there were no significant differences in clinicopathologic characteristics or the *BRAF* mutational rate between the two groups. *Conclusions*. Patients with WLPTC have similar demographic, clinical, pathologic, and molecular characteristics to those with classic PTC coexisting with Hashimoto's thyroiditis.

## 1. Introduction

Papillary thyroid carcinoma (PTC) is the most common malignant neoplasm of the thyroid gland and many variants of PTC have been described including classic, follicular, tall cell, diffuse sclerosing, solid, oncocytic, columnar cell, cribriform-morular, and clear cell variants [1]. Most PTCs have a good prognosis, but some PTC subtypes such as tall cell, columnar cell, and hobnail variants have a more aggressive clinical course [1–3]. The Warthin-like variant of PTC (WLPTC) is a rare variant of PTC that is considered to be a subtype of the oncocytic variant [1]. It has papillary or follicular structures lined by oncocytic cells showing typical PTC nuclear features and marked lymphoplasmacytic infiltration of the stroma. Since WLPTC was first described in 1995, it has generally been considered having similar or less aggressive clinical behavior to classic PTC [4–6]. However, published studies on WLPTC include only a few case reports with short follow-up, and the pathological characteristics and clinical behavior of WLPTCs have not been well documented.

WLPTCs are commonly associated with lymphoid infiltration in tumoral and nontumoral areas, and some authors considered this an implication of favorable prognosis [7]. Recent studies on the coexistence of Hashimoto's thyroiditis (HT)/chronic lymphocytic thyroiditis in PTCs have reported less aggressive pathologic features with better long-term outcome than PTCs without HT [8–10]. Thus, we postulated that WLPTC, frequently accompanied by HT, might have a different prognosis from classic PTC. To date, there has not been a study comparing the clinicopathologic behavior of WLPTC with that of classic PTC. The aim of this study was to investigate the demographic, clinical, and molecular characteristics of patients with WLPTC, classic PTC, and classic PTC with coexisting HT to evaluate prognostic factors for WLPTC.

## 2. Materials and Methods

We performed a retrospective review of database of patients with PTC under approval by the Institutional Review Board of The Catholic University of Korea, Seoul St. Mary's Hospital.

### 2.1. Patients

A total of 2,139 patients underwent surgical treatment and were histologically confirmed to have PTC at Seoul St. Mary Hospital between January 2012 and December 2013. Of these, 40 (1.9%) who were diagnosed with WLPTC were enrolled and 200 consecutive patients with classic PTC were selected as controls. Ipsilateral central neck lymph node dissection was routinely performed in all patients. HT was diagnosed based on histologic findings of the thyroid; peritumoral lymphocytic infiltration alone was excluded. All histologic and cytolopathologic slides were reviewed by three experienced pathologists (Chan Kwon Jung, Min-kyung Yeo, and Youn Soo Lee) with a special interest in thyroid pathology. Consensus was reached on all cases.

### 2.2. Fine Needle Aspiration Cytology

All thyroid fine needle aspiration cytology (FNAC) was performed under ultrasound guidance by experienced radiologists and processed with the ThinPrep preparation method. We classified the FNAC samples according to the Bethesda system for reporting thyroid cytopathology [11].

### 2.3. *BRAF* V600E Mutation Analysis

**Figure 1:**
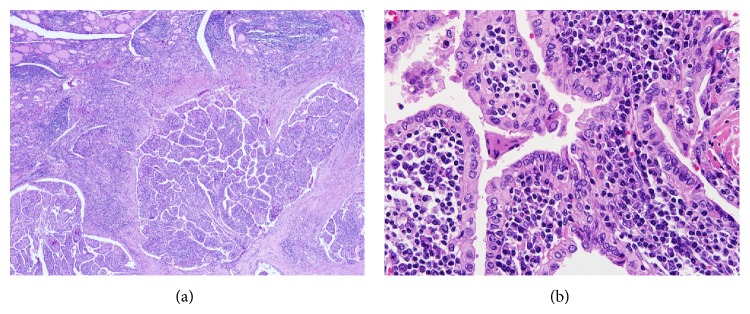
The Warthin-like variant of papillary carcinoma is associated with chronic lymphocytic (Hashimoto) thyroiditis. (a) The tumor shows well-developed papillae with dense lymphoplasmacytic infiltration of the stalks. The surrounding normal thyroid parenchyma exhibits Hashimoto's thyroiditis. (b) Tumor cells have granular oncocytic cytoplasm and nuclear features of papillary carcinoma including optically clear nuclei, nuclear grooves, and intranuclear pseudo-inclusions. A dense infiltration of lymphoplasma cells is seen in the stalk of papillary structures.

Tumor areas were manually microdissected from two or three 10-*μ*m thick deparaffinized tissue sections under a stereomicroscope. Genomic DNA was extracted from the dissected tissue samples using the QIAamp DNA Mini Kit (Qiagen, Hilden, Germany). WLPTC has abundant lymphoplasmacytic infiltration within the tumor (Figure 1). Contamination of a tumor sample by normal cells can lead to false negativity on somatic mutation analysis; therefore, we used pyrosequencing to detect* BRAF* V600E mutation, because pyrosequencing is more sensitive than Sanger sequencing and has similar sensitivity to real-time PCR method [12, 13]. Pyrosequencing was performed on the Pyromark Q24 platform (Qiagen) as described previously [12].

### 2.4. Statistical Analysis

Pearson's chi square and Fisher's exact tests were used to evaluate the relationship between categorical variables, while Student's *t*-tests and Mann-Whitney *U*-tests were used to compare two different groups of continuous parametric data. Statistical analysis was performed with SPSS software (Version 16.0, SPSS, Chicago, IL, USA).

## 3. Results

### 3.1. Clinicopathologic Characteristics of PTC Patients according to Histologic Subtype

The clinicopathologic characteristics of 40 WLPTC and 200 classic PTC patients are summarized in Table 1. The median age at surgery for WLPTC and classic PTC patients was 46 years and 45 years, respectively, with no significant difference between groups. The WLPTC patients had larger tumor size (median: 1.0 cm, ranged from 0.4 to 2.2 cm) than classic PTC patients (median: 0.7 cm) (*P* = 0.043). HT more frequently coexisted with WLPTC than classic PTC (93% versus 36%, resp., *P* < 0.001). There were no significant differences in multifocality, extrathyroid extension, pathologic (p) T stage, lymph node metastasis, or preoperative diagnosis between the two groups (Table 1). However, the* BRAF* V600E mutation rate was significantly lower in WLPTC than in classic PTC (65% versus 84%, resp., *P* = 0.015). Classic PTC coexisting with HT showed a significantly lower* BRAF* mutational rate than that without HT (74% versus 87%, resp., *P* = 0.031). However, there were no significant differences in clinicopathologic features between classic PTC patients with or without HT (Table 2).

### 3.2. Clinicopathologic Characteristics of WLPTC and Classic PTC in Patients with Coexisting Hashimoto's Thyroiditis

When we compared WLPTC and classic PTC in patients with coexisting Hashimoto's thyroiditis, there were no significant differences in age, sex, multifocality, tumor size, pT stage, extrathyroidal extension, lymph node metastasis, preoperative diagnosis, or* BRAF* V600E mutation (Table 3).

### 3.3. Clinicopathologic Characteristics of Microcarcinomas (≤1.0 cm in Size) according to Histologic Subtype

There were no significant differences in age, sex, multifocality, pT stage, extrathyroidal extension, lymph node metastasis, preoperative diagnosis, or* BRAF* V600E mutation (Table 4). WLPTC microcarcinoma patients had larger tumor size (mean: 0.7 cm) than classic PTC microcarcinoma patients (mean: 0.6 cm) (*P* = 0.001). HT more frequently coexisted with WLPTC than with classic PTC (96% versus 35%, resp., *P* < 0.001).

## 4. Discussion

**Figure 2:**
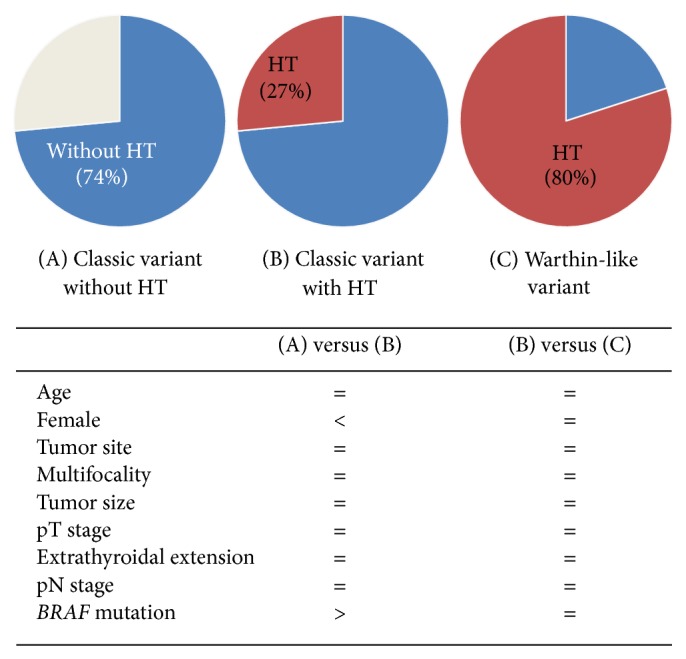
A schematic diagram summarizing the relationship between the Warthin-like variant of papillary carcinoma and classic papillary carcinomas with or without Hashimoto's thyroiditis according to clinicopathologic features.

The presence of HT in PTC has been associated with favorable prognostic features, such as lower rates of lymph node metastasis, extrathyroidal extension, and TNM stage, and lower frequency of* BRAF* V600E mutation [8, 9, 14–18]. The peculiar lymphoid infiltrates in the papillary stalks suggest that WLPTC might have a distinguished entity [7]. WLPTC is commonly accompanied by HT in a background [19]. In the present study, HT was seen in 80% of all WLPTC cases. We hypothesized that WLPTC might have better prognosis than classic PTC due to an association with HT. However, there were no significant differences in clinicopathologic factors (age, sex, multifocality, pT stage, extrathyroid extension, and lymph node metastasis) except for tumor size, HT, and* BRAF* mutation between WLPTC and classic PTC. When we compared WLPTC and classic PTC with HT only, there were no significant differences in age, sex, multifocality, pT stage, extrathyroid extension, lymph node metastasis, or even tumor size or* BRAF* V600E mutation between groups. Thus, we suggest that the pathologic and clinical behaviors of WLPTCs are similar to those of classic PTC, especially classic PTC with HT (Figure 2).

Despite the fact that* BRAF* mutations have been found in >80% of classic PTC [20], data on mutation frequencies are only available from a small series of WLPTCs: 6/8 (75%), 2/2 (100%), 2/3 (67%), and 0/3 (0%) [21–24]. In our study, the* BRAF* V600E was found in 26 (65%) of 40 patients with WLPTC. There was no relationship between* BRAF* V600E status and clinicopathologic features (age, sex, tumor size, multifocality, extrathyroidal extension, pT stage, and lymph node metastasis) in WLPTC (data not shown).

**Figure 3:**
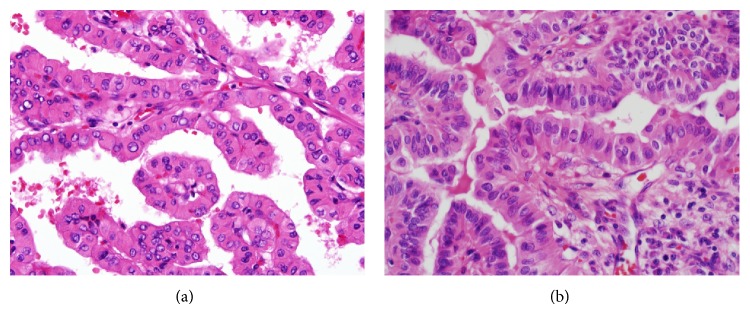
Differential diagnosis of the Warthin-like variant of papillary carcinoma includes oncocytic and tall cell variants. (a) The oncocytic variant has eosinophilic granular cytoplasm. (b) The tall cell variant contains tall cells with a height at least twice their width and eosinophilic cytoplasm. Dense stromal lymphoplasmacytic infiltration is absent in both oncocytic and tall cell variants.

The prevalence of WLPTC was 1.9% (40/2, 139) of all PTCs in our study population. However, it seems that the incidence has been underestimated because WLPTCs are often misclassified as classic, tall cell, or oncocytic variants [3]. In a recent Korean study, Jun et al. reported 16 (0.2%) WLPTCs of 8,179 PTCs [25]. Although classic PTC and WLPTC show a similar growth pattern, the papillary cores of the classic variant do not show marked infiltration of lymphocytes and plasma cells [3, 26]. Because tumor cells of WLPTC have abundant eosinophilic cytoplasm, the differential diagnosis includes tall cell and oncocytic (Hűrthle cell) variants of PTC (Figure 3). The tall cell variant consists of ≥50% tall cells. Tall cells have a height at least twice their width with a dense eosinophilic cytoplasm and prominent cell borders [3, 20, 27]. Tall cell variants often exhibit closely packed papillae and thin elongated follicles [20]. In the oncocytic variant, tumor cells show dense eosinophilic, granular cytoplasm and the typical nuclear features of PTC [3]. In tall cell and oncocytic variants, papillary structures do not have dense infiltration of lymphoplasma cells in their stalks.

**Figure 4:**
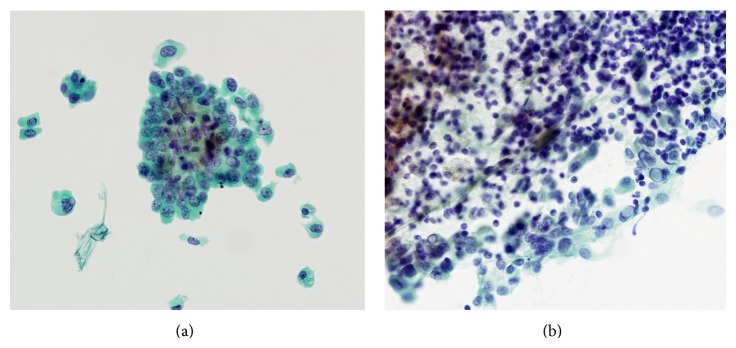
ThinPrep liquid-based cytology and conventional smear of the Warthin-like variant of papillary carcinoma. (a) ThinPrep cytology shows higher cellularity and clean background compared to conventional smear. (b) In the conventional smear, there was dense lymphoplasmacytic infiltration in the cellular clusters.

The cytologic diagnosis of WLPTC may be difficult due to an abundant lymphoid background and oncocytic change in tumor cells [28]. In our study, there was no difference in FNAC results between classic PTCs and WLPTCs. As the ThinPrep method filters inflammatory cells in thyroid FNAC samples, obscured lymphocytes and plasma cells are markedly decreased in the liquid-based preparation when compared with conventional smears (Figure 4). Thus, cytomorphology could be easily detected in WLPTCs using ThinPrep.

The limitations of our study include its retrospective design and lack of follow-up to estimate patient prognosis.

In conclusion, patients with WLPTC had similar demographic, clinical, pathologic, and molecular characteristics to those with classic PTC coexisting with HT. Further long-term follow-up studies are necessary to confirm the biologic behavior and prognosis of WLPTC.

## Figures and Tables

**Table 1:** Comparison of clinicopathologic characteristics between WLPTC and classic PTC.

Characteristic	Classic PTC (*N* = 200)	WLPTC (*N* = 40)	*P* value
Mean age (years)	45 (22–73)	46 (18–77)	0.595
Age			0.729
<45	94 (47%)	17 (43%)	
≥45	106 (53%)	23 (58%)	
Sex			0.374
Female	161 (81%)	35 (88%)	
Male	39 (20%)	5 (13%)	
Multifocality			0.385
Unifocal	108 (54%)	25 (63%)	
Multifocal	92 (46%)	15 (38%)	
Surgery type			0.088
Lobectomy	63 (32%)	7 (18%)	
Total thyroidectomy	137 (69%)	33 (83%)	
Median tumor size (cm)	0.7 (0.2–4.5)	1.0 (0.4–2.2)	0.043
pT stage			0.134
pT1	123 (62%)	30 (75%)	
pT2	2 (1%)	1 (3%)	
pT3	75 (38%)	9 (23%)	
Extrathyroidal extension			0.073
Absent	125 (63%)	31 (78%)	
Present	75 (38%)	9 (23%)	
pN stage			0.730
pN0	102 (51%)	22 (55%)	
pN1	98 (49%)	18 (45%)	
Hashimoto's thyroiditis			<0.001
Absent	147 (74%)	8 (20%)	
Present	53 (27%)	32 (80%)	
Preoperative diagnosis			0.697
Atypia of undetermined significance	7 (4%)	2 (5%)	
Suspicious for PTC	45 (23%)	7 (18%)	
PTC	148 (74%)	31 (78%)	
*BRAF* mutation			0.015
Absent	33 (17%)	14 (35%)	
Present	167 (84%)	26 (65%)	

PTC, papillary thyroid carcinoma; WLPTC, Warthin-like variant of papillary thyroid carcinoma.

**Table 2:** Comparison of clinicopathologic characteristics between classic PTC with or without HT.

Characteristic	Classic PTC with HT (*N* = 53)	Classic PTC without HT (*N* = 147)	*P* value
Median age (years)	46 (25–73)	45 (22–73)	0.357
Age			0.261
<45	21 (40%)	73 (50%)	
≥45	32 (60%)	74 (50%)	
Sex			<0.001
Female	52 (98%)	109 (74%)	
Male	1 (2%)	38 (26%)	
Multifocality			0.264
Single	25 (47%)	83 (57%)	
Multiple	28 (53%)	64 (44%)	
Surgery type			0.122
Lobectomy	12 (23%)	51 (35%)	
Total thyroidectomy	41 (77%)	96 (65%)	
Median tumor size (cm)	0.7 (0.3–4.5)	0.6 (0.2–3.3)	0.343
pT stage			0.532
pT1	34 (64%)	89 (61%)	
pT2	1 (2%)	1 (1%)	
pT3	18 (34%)	57 (39%)	
Extrathyroidal extension			0.620
Absent	35 (66%)	90 (61%)	
Present	18 (34%)	57 (39%)	
pN stage			0.262
pN0	31 (59%)	71 (48%)	
pN1	22 (42%)	76 (52%)	
Preoperative diagnosis			0.904
Atypia of undetermined significance	2 (4%)	5 (3%)	
Suspicious for PTC	13 (25%)	32 (22%)	
PTC	38 (72%)	110 (75%)	
*BRAF* mutation			0.031
Absent	14 (26%)	19 (13%)	
Present	39 (74%)	128 (87%)	

PTC, papillary thyroid carcinoma; WLPTC, Warthin-like variant of papillary thyroid carcinoma; HT, Hashimoto's thyroiditis.

**Table 3:** Comparison of clinicopathologic characteristics between WLPTC and classic PTC coexisting with HT.

Characteristic	Classic PTC with HT (*N* = 53)	WLPTC (*N* = 40)	*P* value
Mean age (years)	46 (25–73)	46 (18–77)	0.892
Age			0.833
<45	21 (40%)	17 (43%)	
≥45	32 (60%)	23 (58%)	
Sex			0.081
Female	52 (98%)	35 (88%)	
Male	1 (2%)	5 (13%)	
Multifocality			0.207
Unifocal	25 (47%)	25 (63%)	
Multifocal	28 (53%)	15 (38%)	
Surgery type			0.611
Lobectomy	12 (23%)	7 (18%)	
Total thyroidectomy	41 (77%)	33 (83%)	
Median tumor size (cm)	0.7 (0.3–4.5)	1.0 (0.4–2.2)	0.285
pT stage			0.467
pT1	34 (64%)	30 (75%)	
pT2	1 (2%)	1 (3%)	
pT3	18 (34%)	9 (23%)	
Extrathyroidal extension			0.257
Absent	35 (66%)	31 (78%)	
Present	18 (34%)	9 (23%)	
pN stage			0.833
pN0	31 (59%)	22 (55%)	
pN1	22 (42%)	18 (45%)	
Preoperative diagnosis			0.675
Atypia of undetermined significance	2 (4%)	2 (5%)	
Suspicious for PTC	13 (25%)	7 (18%)	
PTC	38 (72%)	31 (78%)	
*BRAF* mutation			0.494
Absent	14 (26%)	14 (35%)	
Present	39 (74%)	26 (65%)	

PTC, papillary thyroid carcinoma; WLPTC, Warthin-like variant of papillary thyroid carcinoma; HT, Hashimoto's thyroiditis.

**Table 4:** Comparison of clinicopathologic characteristics between WLPTC and classic PTC microcarcinomas (≤1 cm).

Characteristics of microcarcinomas	Classic PTC (*N* = 161)	WLPTC (*N* = 25)	*P* value
Mean age (years)	45 (23–73)	44 (18–63)	0.871
Age			1.000
<45	75 (47%)	12 (48%)	
≥45	86 (53%)	13 (52%)	
Sex			0.263
Female	129 (80%)	23 (92%)	
Male	32 (20%)	2 (8%)	
Multifocality			0.196
Unifocal	93 (58%)	18 (72%)	
Multifocal	68 (42%)	7 (28%)	
Surgery type			0.378
Lobectomy	62 (39%)	7 (28%)	
Total thyroidectomy	99 (62%)	18 (72%)	
Median tumor size (cm)	0.6 (0.2–1.0)	0.7 (0.4–1.0)	0.001
pT stage			0.476
pT1	116 (72%)	20 (80%)	
pT2	0 (0%)	0 (0%)	
pT3	45 (28%)	5 (20%)	
Extrathyroidal extension			0.476
Absent	116 (72%)	20 (80%)	
Present	45 (28%)	5 (20%)	
pN stage			0.385
pN0	92 (57%)	17 (68%)	
pN1	69 (43%)	8 (32%)	
Hashimoto's thyroiditis			<0.001
Absent	121 (75%)	5 (20%)	
Present	40 (25%)	20 (80%)	
Preoperative diagnosis			0.521
Atypia of undetermined significance	6 (4%)	2 (8%)	
Suspicious for PTC	40 (25%)	6 (24%)	
PTC	114 (71%)	17 (68%)	
*BRAF* mutation			0.103
Absent	28 (17%)	8 (32%)	
Present	133 (83%)	17 (68%)	

PTC, papillary thyroid carcinoma; WLPTC, Warthin-like variant of papillary thyroid carcinoma.
